# The Skeletal Amino Acid Composition of the Marine Demosponge *Aplysina cavernicola*

**DOI:** 10.3390/md12084417

**Published:** 2014-08-08

**Authors:** Susanne Ueberlein, Susanne Machill, Hendrik Niemann, Peter Proksch, Eike Brunner

**Affiliations:** 1 Bioanalytical Chemistry, TU Dresden, Dresden 01062, Germany; E-Mails: susanne.ueberlein@chemie.tu-dresden.de (S.U.); susanne.machill@chemie.tu-dresden.de (S.M.); 2 Institute of Pharmaceutical Biology and Biotechnology, Heinrich Heine University Düsseldorf, Universitaetsstrasse 1, Geb. 26.23, Düsseldorf 40225, Germany; E-Mails: hendrik.niemann@uni-duesseldorf.de (H.N.); proksch@uni-duesseldorf.de (P.P.)

**Keywords:** demosponges, skeletons, amino acid composition, halogenated amino acids, GC-MS, LC-MS

## Abstract

It has been discovered during the past few years that demosponges of the order Verongida such as *Aplysina cavernicola* exhibit chitin-based skeletons. Verongida sponges are well known to produce bioactive brominated tyrosine derivatives. We could recently demonstrate that brominated compounds do not exclusively occur in the cellular matrix but also in the skeletons of the marine sponges *Aplysina cavernicola* and *Ianthella basta*. Our measurements imply that these yet unknown compounds are strongly, possibly covalently bound to the sponge skeletons. In the present work, we determined the skeletal amino acid composition of the demosponge *A. cavernicola* especially with respect to the presence of halogenated amino acids. The investigations of the skeletons before and after MeOH extraction confirmed that only a small amount of the brominated skeleton-bound compounds dissolves in MeOH. The main part of the brominated compounds is strongly attached to the skeletons but can be extracted for example by using Ba(OH)_2_. Various halogenated tyrosine derivatives were identified by GC-MS and LC-MS in these Ba(OH)_2_ extracts of the skeletons.

## 1. Introduction

Sponges (*Porifera*) belong to the oldest *Metazoans* and are the simplest animals on earth [1,2]. Although they do not possess morphological defense strategies, these sessile filter feeders successfully withstand the attacks of predators, as well as overgrowth by fouling organisms or bacterial infections. This evolutionary success is mainly due to an effective chemical defense based on deterrent, cytotoxic and/or antibiotically active compounds [3,4,5,6].

Sponges possess a skeleton which consists of a composite of natural biomaterials containing organic constituents and/or inorganic compounds. One function of these skeletons is mechanical support, that means the prevention of the collapse of the sponge body [7,8]. Furthermore, the skeletons seem to play a role in the protection against predators. For example, Hill *et al.* showed that the sponge *Anthosigmella varians* increases the spicule concentration during the attack of predators [9,10].

Based on the skeletal composition, the phylum *Porifera* is divided into three classes: Calcareous sponges (*Calcarea*), glass sponges (*Hexactinellida*), and demosponges (*Demospongia*) [11]. Demosponges form the largest class and possess a skeleton made of a composite material with different organic compounds like proteins [12] and polysaccharides [13,14]. They can also contain siliceous spicules [11,15]. The skeletons of three taxonomic orders of demosponges (Dendroceratida, Dictyoceratida, Verongida) are characterized by the lack of siliceous spicules. Instead, their skeletons contain spongin, a collagenous protein [16].

Until now, the chemical composition of spongin has not been exactly defined. Gross *et al.* isolated two morphologically distinct forms of spongin described as “spongin A” and “spongin B” [17]. Spongin fibers are more resistant than collagen fibers against enzymatic digestion [18]. Today, spongin is characterized as a halogenated protein [16]. Ehrlich *et al.* demonstrated recently that the polysaccharide chitin is an integral part of the skeleton of different demosponges of the order Verongida [13,14,19]. So far, chitin could be found in the skeletons of the genus *Aplysina* [13,19], in the skeletons of *Verongula gigantea* [19], *Ianthella basta* [14], and the freshwater demosponge *Spongilla lacustris* [20]. Moreover, chitin could be isolated from a million year old fossil of the basal demosponge *Vauxia gracilenta* [21].

It is unknown yet how the organic components such as proteins (spongin) are connected with the chitin in sponge skeletons [16]. However, there is a variety of examples in nature showing that proteins are strongly bound to chitin. For example, Hackman suggested that proteins are covalently bound to chitin in different insects and crustacea [22]. Blackwell *et al.* developed a model for the three-dimensional structure of an insect chitin-protein complex with chitin fibrils surrounded by layers of proteins [23].

Furthermore, Verongida sponges such as *A. cavernicola* are well-known for the biosynthesis of brominated tyrosine derivatives—characteristic bioactive natural products [24,25]. Up to now it has remained unclear whether these compounds are exclusively present in the cellular matrix or whether they may also be incorporated into the chitin-based skeletons. Earlier investigations showed that aerothionin, a typical brominated tyrosine-derivative, is present in the spherulous cells of the marine sponge *Aplysina fistularis* [26]. Another earlier report demonstrated that the brominated compounds are localized also in the skeletal fibers of the demosponge *Aplysina aerophoba* [27]. We recently demonstrated that brominated compounds occur not exclusively in the cellular matrix but also in the skeletons of the marine sponges *A. cavernicola* and *I. basta*. The bromine content of the skeletons of these sponges was measured by quantitative potentiometric titration. A MeOH treatment only resulted in the removal of rather insignificant amounts of bromine. It was therefore concluded that these yet unknown brominated compounds are strongly, possibly covalently bound to the chitin-based sponge skeletons [28]. The structure and composition of these compounds is yet unknown. One possibility could be a covalent incorporation of halogenated amino acids into the skeletons.

Until now the pure skeletal amino acid composition of the chitin-containing Verongida sponges has been completely unknown since only the amino acid compositions of the commercial unbleached sponge *Hippospongia equina* and of the bath sponge *Spongia officinalis obliqua* which both belong to the order Dictyoceratida have been investigated [29,30]. The analyses of these sponges indicated that not only proteinogenic amino acids but also halogenated tyrosines occur in these sponges. Iodated tyrosines were found in *Hippospongia equina* [29], while iodated as well as brominated tyrosines could be detected in *Spongia officinalis obliqua* [30].

In contrast to these previous studies, the goal of the present study was to examine the pure skeletal amino acid composition of the Verongida sponge *A. cavernicola*, especially with respect to halogenated compounds. We therefore isolated the sponge skeletons as described previously [28]. Furthermore, we developed a method to analyze the amino acid composition of the skeletons. In order to evaluate the influence of the established MeOH extraction upon the skeletal amino acid composition, the sponge skeletons were studied before and after MeOH extraction. MeOH is widely used for the extraction of bioactive natural products like aerothionin [31]. In addition, the MeOH extract was analyzed in order to identify possible MeOH-extractable soluble components.

## 2. Results and Discussion

### 2.1. Examination of the Skeletons by SEM

**Figure 1:**
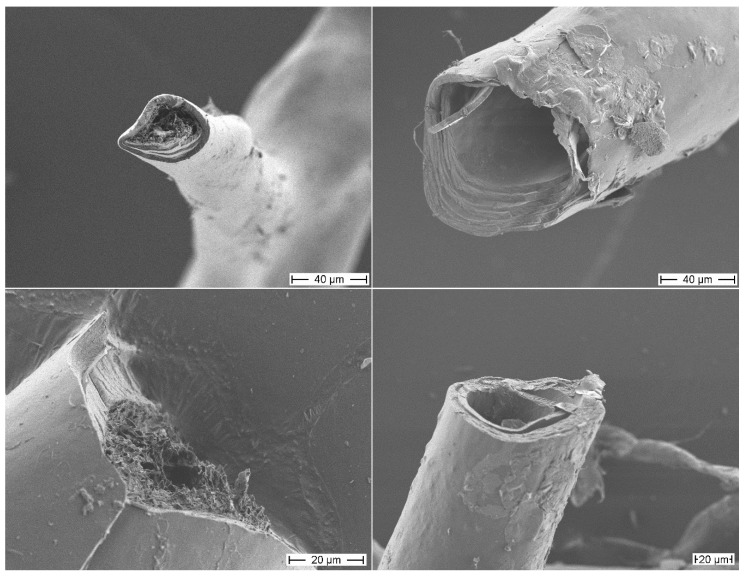
Scanning electron microscopy (SEM) images of the skeletons of *A. cavernicola* before MeOH extraction (left) and after MeOH extraction (right), scale bars: 40 μm (top), 20 μm (bottom).

The morphology of the isolated sponge skeletons before and after MeOH treatment was studied by scanning electron microscopy (SEM) (Figure 1). The SEM image of the isolated skeletons before MeOH treatment shows a skeletal fiber network system with network fibers composed of concentric multilayers. The core channel of these fibers is filled with a porous material, a pith. The SEM images of the skeletons after MeOH extraction show the same network of fibers with concentric multilayered channels. However, the porous core material is removed by the MeOH treatment showing that this material is only loosely attached to the inner layer surface. This MeOH-soluble pith material represents only 1.4% of the total mass of the isolated sponge skeleton.

Moreover, subsequent energy dispersive X-ray spectroscopy (EDX) measurements show that bromine, chlorine, and iodine are present in the skeletons before as well as after MeOH extraction (see supporting information Figures S1 and S2). This is in line with our aforementioned hypothesis that halogenated compounds are tightly attached/covalently bound to the sponge skeletons.

### 2.2. Amino Acid Extraction, Selection of the Derivatization Agent and the Internal Standard for GC-MS

**Figure 2:**
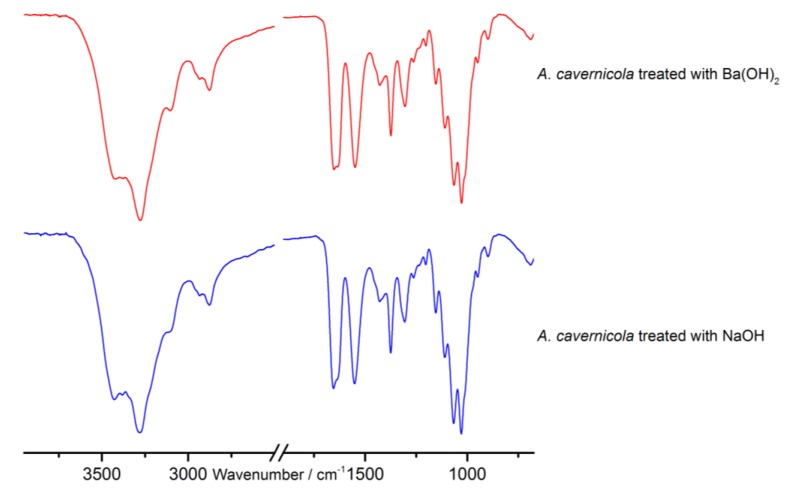
Attenuated total reflectance infrared (ATR-IR) spectra of the purified chitin-scaffolds of *A.* *cavernicola* after Ba(OH)_2_ and NaOH treatment.

**Figure 3:**
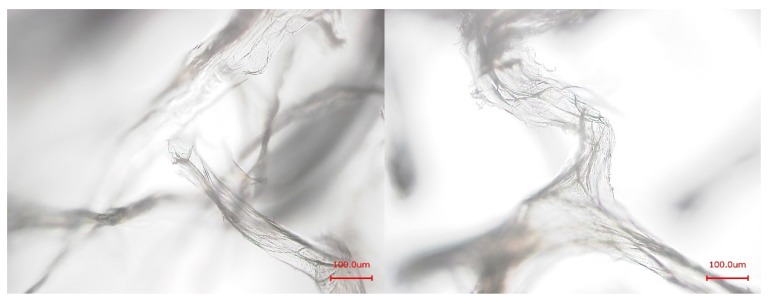
Light microscopic images of the purified chitin-scaffolds of *A.* *cavernicola* after Ba(OH)_2_ (left) and NaOH treatment (right); scale bars: 100 μm.

Alkaline extraction of pure chitin scaffolds from marine sponges using NaOH as previously described by Ehrlich *et al.* [19] leads to a degradation and removal of all other skeleton-associated or incorporated biomolecules such as brominated tyrosine-derivatives or proteins like spongin—but leaves the chitin undissolved. The proteins are hydrolyzed, e.g., disintegrated into their amino acids by this treatment. These extracts, however, contain a huge amount of NaOH which interferes with the further analytical procedure. In order to circumvent this problem, a saturated Ba(OH)_2_ solution was used as extracting and hydrolyzing agent. Subsequently it was investigated whether the Ba(OH)_2_ treatment had the same effect as the NaOH treatment. ATR-FTIR measurements (Figure 2) and microscopic studies (Figure 3) show that the Ba(OH)_2_ as well as the NaOH extraction lead to the removal of all other organic material (cf. [28]) apart from the pure chitin scaffolds. The remaining chitin represents 8.0% ± 1.4% of the mass of the isolated sponge skeleton. In contrast to NaOH, however, the Ba(OH)_2_ extract can be easily neutralized by adding H_2_SO_4_. The resulting BaSO_4_ forms a precipitate which is separated by centrifugation whereas the extracted amino acids and other soluble compounds remain in the supernatant. After freeze drying, these extracted compounds can be identified by gas chromatography-mass spectrometry (GC-MS). The latter technique requires a preceding derivatization of the compounds.

For this purpose, the derivatization agent *N*-*tert*-butyldimethylsilyl-*N*-methyltrifluoroacetamide (MTBSTFA) containing 1% *tert*-butyldimethylchlorosilane (TBDMSCl) was selected since *tert*-butyldimethylsilyl (TBDMS) derivatives of amino acids are more stable than the traditional trimethylsilyl (TMS) derivatives [32]. The amino acids were identified based on their mass spectra and—If available—By comparison with standards. Therefore, the relative retention times of the amino acids found in the extract were compared with the pure reference compounds (standards). The compound 5-Bromotryptophan was selected as an internal standard due to its similarity with the halogenated compounds found in the skeletons, *i.e.*, the brominated aromatic ring system. This internal standard shows two GC-signals due to different degrees of derivatization (first peak: two TBDMS groups; second peak: three TBDMS groups).

### 2.3. GC-MS Analysis of the Skeletal Amino Acid Composition

#### 2.3.1. Skeletal Amino Acid Composition before MeOH Extraction

**Figure 4:**
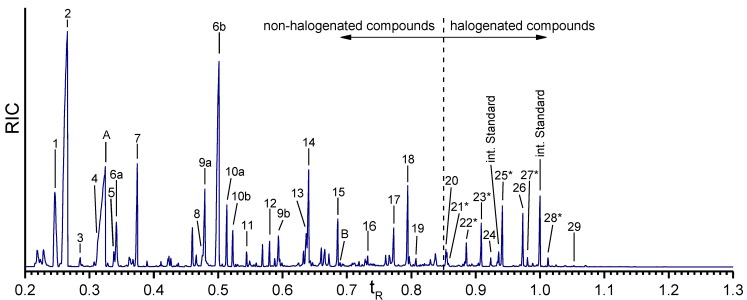
Gas chromatogram of the Ba(OH)_2_ extract of the isolated *A.* *cavernicola* sponge skeletons before MeOH treatment. The relative retention time is related to the second peak of the internal standard. Components with * could not be verified with pure standards due to the lack of availability of these reference compounds.

The isolated skeletons [28] of the demosponge *A. cavernicola* were treated with a saturated Ba(OH)_2_ solution and derivatized using MTBSTFA as described in Section 2.2 and in the Experimental Section. The GC-MS measurements of the isolated *A. cavernicola* sponge skeletons show the presence of various amino acids (see Table 1 and Figure 4).

**Table 1:** Amino acids detected in the Ba(OH)_2_ extract of isolated *A.* *cavernicola* sponge skeletons before MeOH treatment. Components with * could not be verified with pure standards due to the lack of availability of these reference compounds.

Peak	*tert*-Butyldimethylsilyl (TBDMS)-Derivative	Proteinogenic	Halogenated
1	Alanine	X	
2	Glycine	X	
3	α-Aminobutyric Acid (AABA)		
4	Valine	X	
A	Urea		
5	Leucine	X	
6a	Serine (2 TBDMS)	X	
7	Proline	X	
8	Oxoproline		
9a	Hydroxyproline (2 TBDMS)		
6b	Serine (3 TBDMS)	X	
10a	Threonine (3 TBDMS)	X	
10b	Threonine (3 TBDMS)	X	
11	Phenylalanine	X	
12	Aspartic Acid	X	
9b	Hydroxyproline (3 TBDMS)		
13	Glutamic Acid	X	
14	Ornithine		
15	Lysine	X	
B	Aerothionin or its derivatives		
16	Arginine	X	
17	Histidine	X	
18	Tyrosine	X	
19	Tryptophan	X	
20	3-Monochlorotyrosine		X
21*	Monobromohistidine		X
22*	Monobromotyrosine		X
23*	Dichlorotyrosine		X
24	3-Monoiodotyrosine		X
25*	Monobromo-monochlorotyrosine		X
26	3,5-Dibromotyrosine		X
27*	Monochloro-monoiodotyrosine		X
28*	Monobromo-monoiodotyrosine		X
29	3,5-Diiodotyrosine		X

Nineteen different non-halogenated amino acids elute below a relative retention time of *t*_R_ = 0.85. Fifteen of them are proteinogenic amino acids. Several amino acids give rise to multiple peaks. Serine (peaks 6a, 6b) and hydroxyproline (peaks 9a, 9b) exhibit two signals due to different degrees of derivatization. Threonine (peaks 10a, 10b) yields two peaks for the same TBDMS derivative in the extracts as well as in the standard. Due to the existence of two stereocenters, threonine (peaks 10a, 10b) exhibits two different diastereomers [33] which can be separated by gas chromatography.

The largest peaks in the chromatogram are due to glycine (peak 2) and serine (peaks 6a, 6b). Moreover, proline (peak 7), threonine (peaks 10a, 10b), and tyrosine (peak 18) occur in larger quantities, while aspartic acid (peak 12), glutamic acid (peak 13), lysine (peak 15), and histidine (peak 17) occur in smaller amounts only. Leucine (peak 5), phenylalanine (peak 11), arginine (peak 16), and tryptophan (peak 19) exhibit the smallest concentrations. The amount of valine (peak 4) cannot be determined exactly due to the overlap with the urea peak. Nevertheless, an evaluation of specific ion tracks belonging to valine is possible and indicates a low concentration. Furthermore, the non-proteinogenic amino acids α-aminobutyric acid (peak 3), oxoproline (peak 8), hydroxyproline (peaks 10a, 10b), and ornithine (peak 14) can be identified. Measurements of the arginine standard treated with Ba(OH)_2_ under the same conditions as the skeletons reveal that one part of arginine is cracked into urea (peak A) and ornithine by the alkaline treatment. This leads to the assumption that the detected amount of urea in the Ba(OH)_2_ extracts of the sponge skeletons may arise from the Ba(OH)_2_ treatment of arginine. However, the comparison of the arginine/ornithine and arginine/urea ratios in the standard and the sample of the skeletons (see Table 2) reveal that the relative amounts of ornithine and urea related to arginine are larger in the Ba(OH)_2_ extract of the sponge skeletons than in the standard. This leads to the assumption that urea and ornithine are indeed present in the sponge skeletons and are not just artefacts of the Ba(OH)_2_ treatment.

**Table 2:** Comparison of the arginine/ornithine and arginine/urea ratios calculated for the standard and the skeleton sample before MeOH treatment.

Sample	Arginine/Ornithine Proportion	Arginine/Urea Proportion
Standard: arginine after Ba(OH)2	1:2.6	1:1.1
Ba(OH)2 extract of sponge skeletons before MeOH	1:8.8	1:9.1

Ten halogenated amino acids elute at relative retention times larger than 0.85. Interestingly, nine of them are tyrosine derivatives. Monohalogenated (e.g., monobromotyrosine (peak 22*)), dihalogenated (e.g., dichlorotyrosine (peak 23*)) as well as mixed halogenated (e.g., monobromo-monoiodotyrosine (peak 28*)) tyrosine occur. Only one halogenated derivative of another amino acid is identified, a monobrominated histidine (peak 21*). Dichlorotyrosine (peak 23*), monobromo-monochlorotyrosine (peak 25*), and 3,5-dibromotyrosine (peak 26) represent the most abundant halogenated amino acids. Monobromotyrosine (peak 22*) also occurs at relatively high concentration. The other halogenated amino acids occur only in small amounts.

**Figure 5:**
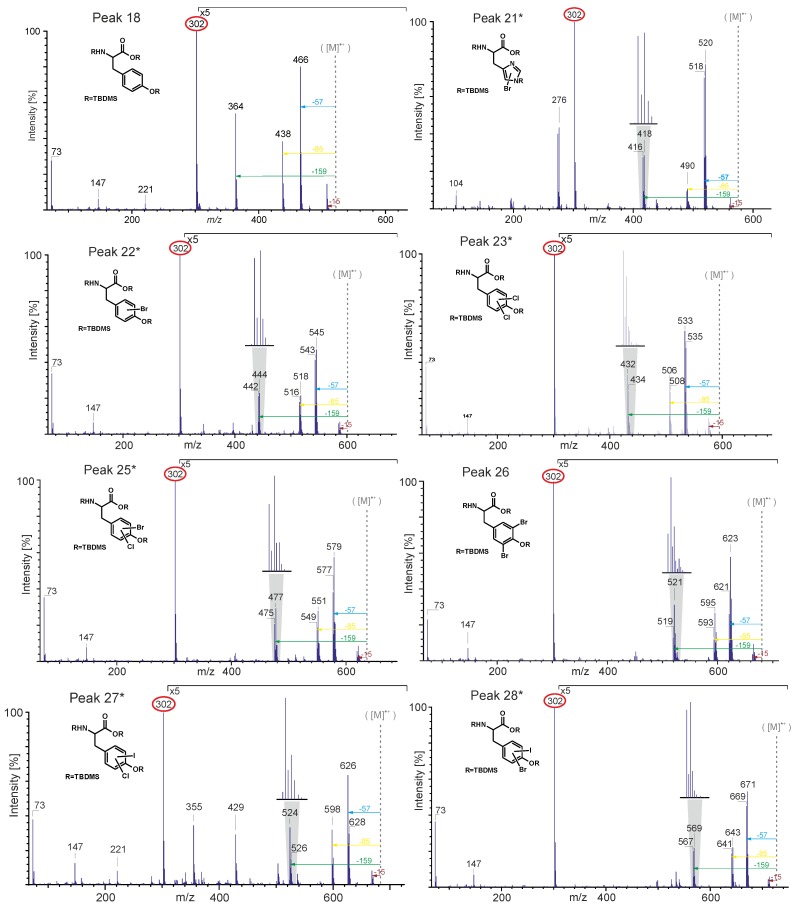
Electron impact (EI)-MS data of TBDMS-derivatives of tyrosine (peak 18), monobromohistidine (peak 21*), monobromotyrosine (peak 22*), dichlorotyrosine (peak 23*), monobromo-monochlorotyrosine (peak 25*), 3,5-dibromotyrosine (peak 26), monochloro-monoiodotyrosine (peak 27*) and monobromo-monoiodotyrosine (peak 28*).

Due to the lack of pure standard compounds, most of the halogenated amino acids were identified by their electon impact (EI) mass spectra. Figure 5 shows the EI mass spectra of the TBDMS derivatives of tyrosine and various halogenated tyrosines. The theoretical molecular weights of the expected compounds were calculated and compared with the experimentally observed fragmentation patterns in the mass spectra. Usually, the molecular ion peak is very weak or missing. The same is true for the (M-15) peak caused by the loss of a methyl group. Nevertheless, the spectra provide characteristic fragments. In particular, the (M-57) peak due to the loss of a *tert*-butyl group of the TBDMS group is intense in all spectra and can be used to determine the molecular weight of the respective compound. Furthermore, the peaks due to the loss of a *tert*-butyl group and a CO group (M-85) as well as of a CO-O-TBDMS group (M-159) are characteristic and intense. The base peak at *m/z* 302 is due to the [TBDMS-NH-CH-CO-O-TBDMS]^+^-ion representing the backbone of each amino acid.

Brominated and chlorinated compounds could be easily identified by the characteristic isotope patterns due to the presence of two abundant isotopes. Bromine naturally occurs as ^79^Br and ^81^Br with relative abundances of approximately 1:1. Chlorine occurs as ^35^Cl and ^37^Cl with relative abundances of approximately 3:1. This results in characteristic isotope peaks with a mass difference of two mass units [34].

Measurements of a mixture of the standards tyrosine, 3-monoiodotyrosine, 3,5-diiodotyrosine and 3,5-dibromotyrosine treated with Ba(OH)_2_ following the protocol as for the sponge skeletons reveal that the Ba(OH)_2_ treatment has no influence on the structure of the halogenated tyrosines. The GC-MS data show that the Ba(OH)_2_ treatment did not cause any chemical transformation of the amino acids. It can, therefore, be assumed that all detected tyrosine derivatives occur in the native sponge skeletons.

The comparison of the observed amino acid composition of *A. cavernicola* with the known amino acid compositions of different sponges shows a general agreement—but also some characteristic differences. The results of these investigations are summarized in Table 3. The extraction of the amino acids from *Hippospongia equina* [29] and the bath sponge *Spongia officinalis obliqua* [30] was also based on an alkaline solution of Ba(OH)_2_, *i.e.*, the data are well comparable. The observed differences in the amino acid compositions may be related to the fact that both, *H. equina* and the bath sponge, belong to the order Dictyoceratida. In contrast, *A. cavernicola* belongs to the order Verongida.

**Table 3:** Comparison of the found amino acids with the known amino acid composition of spongin from the literature.

Amino Acids (AAs)	Present Work	AAs in *Hippospongia equina* [29]	AAs in Spongia *Officinalis obliqua* [30]
α-Aminobutyric Acid (AABA)	X		X
γ-Aminobutyric Acid (GABA)		X	
Alanine	X	X	X
Arginine	X	X	
Aspartic Acid	X	X	X
Cystine		X	
Glutamic Acid	X	X	X
Glycine	X	X	X
Histidine	X	X	
Hydroxyproline	X	X	X
Leucine	X	X	X
Lysine	X	X	X
Methionine			X
Ornithine	X	X	
Oxoproline	X		
Phenylalanine	X	X	
Proline	X	X	X
Serine	X	X	
Threonine	X	X	
Tryptophan	X	X	X
Tyrosine	X	X	X
Valine	X	X	X
Monobromohistidine	X		
Monobromotyrosine	X		
3-Monochlorotyrosine	X		
3-Monoiodotyrosine	X		X
Monochloro-monoiodotyrosine	X		
Monobromo-monochlorotyrosine	X		
Monobromo-monoiodotyrosine	X		
Dichlorotyrosine	X		
3,5-Dibromotyrosine	X		X
3,5-Diiodotyrosine	X	X	X

The most striking difference between the amino acid composition of the *A. cavernicola* skeleton and the amino acid compositions described in the literature is the presence of various halogenated amino acids in the *A. cavernicola* sponge skeletons. In particular, the halogenated amino acids monobromotyrosine (peak 22*), monobromo-monochlorotyrosine (peak 25*), 3-monochlorotyrosine (peak 20), dichlorotyrosine (peak 23*), monochloro-monoiodotyrosine (peak 27*), and monobromohistidine (peak 21*) do not occur in *H. equina* and *S. officinalis obliqua*. However, the naturally occurring amino acid monobromo-monochlorotyrosine could already be isolated from the gastropod mollusk *Buccinum undatum* [35]. In addition, the occurrence of AABA (peak 3) or GABA seems to depend on the taxonomy of the investigated sponges. Alanine (peak 1), aspartic acid (peak 12), glutamic acid (peak 13), glycine (peak 2), leucine (peak 5), lysine (peak 15), proline (peak 7), tryptophan (peak 19), tyrosine (peak 18), valine (peak 4) as well as the halogenated amino acid diiodotyrosine (peak 29) are found in both *A. cavernicola* skeletons and the dictyoceratid sponges *H. equina* and *S. officinalis obliqua*. The identified amino acids α-aminobutyric acid (peak 3), hydroxyproline (peaks 9a, 9b), oxoproline (peak 8), and ornithine (peak 14) were also detected previously in *H. equina* and *S. officinalis obliqua*. Both α-aminobutyric acid and hydroxyproline are present in the bath sponge *Spongia officinalis obliqua* [35]. Ornithine and oxoproline were found in *Aplysina aerophoba* [36].

Furthermore, the Ba(OH)_2_ extract of the skeletons was examined with respect to the presence of aerothionin—one major bromotyrosine derivative found in the MeOH extracts of *A. cavernicola* skeletons [28]. Therefore, a standard of aerothionin was derivatized and measured by GC-MS. This measurement shows that aerothionin causes different GC peaks due to the decomposition into smaller fragments during GC injection. In comparison, only the degradation product at *t*_R_ = 0.69 (Peak B in Figure 4) can be found in the Ba(OH)_2_ extract of the skeletons. The retention time, as well as the fragmentation pattern of peak B, are consistent with those of the aerothionin standard. The mass spectrum of this fragment of the standard is shown in the Supplementary Information (Figure S3). Based on the very small intensity of the peak and the lack of the other peaks observed in the standard it can be assumed that aerothionin only occurs in trace amounts. Since other aerothionin derivatives like homoaerothionin, 11-oxoaerothionin, or isofistularin-3 could cause the same fragment occurring at *t*_R_ = 0.69 due to their similar structure, it is not clear whether this peak represents aerothionin only. Its derivatives may also be present as minority components.

#### 2.3.2. Skeletal Amino Acid Composition after MeOH Extraction

The relative amount of each amino acid after MeOH treatment was compared to the amount present before MeOH treatment.

Therefore, all intensities (peak heights) were normalized to the sample weight and the total intensity of both peaks of the internal standard. The comparison of the intensities of each amino acid before and after MeOH treatment enables the estimation of the decrease due to the MeOH extraction. The results are summarized in Table 4.

**Figure 6:**
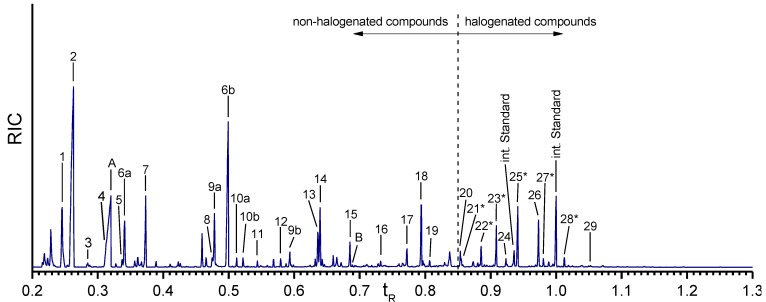
Gas chromatogram of the amino acids in the isolated *A.* *cavernicola* sponge skeletons after MeOH treatment. Retention time is the relative retention time based on the second peak of the internal standard. Components with * could not be verified with standards. Numbering of the peaks is as in Table 1.

The comparison of the GC-MS data of the skeletons after MeOH extraction (Figure 6) with the GC-MS data of the skeletons before MeOH treatment reveals some differences. All amino acids found in the Ba(OH)_2_ extract of the skeletons before MeOH extraction are present. However, their relative amounts have changed. That means, MeOH selectively reduces the amount of several amino acids. Interestingly, the largest decreases are observed for the proteinogenic amino acids. This leads to the assumption that either free amino acids or small proteins/peptides which are not tightly bound to the skeletons are removed by the MeOH extraction.

**Table 4:** Changes of the detected amounts of amino acids after MeOH extraction in comparison with the amounts before MeOH extraction. An average experimental error of approximately 15% is estimated (see Supplementary Information, Table S1).

TBDMS–Derivative	Decrease ^1^
Alanine	+
Glycine	++
α-Aminobutyric Acid (AABA)	+++
Valine	+++
Leucine	+++
Serine (2 TBDMS)	−
Proline	++
Oxoproline	+
Hydroxyproline (2 TBDMS)	++
Serine (3 TBDMS)	++
Threonine (3 TBDMS)	++++
Threonine (3 TBDMS)	++++
Phenylalanine	+++
Aspartic Acid	+++
Hydroxyproline (3 TBDMS)	+++
Glutamic Acid	−
Ornithine	++
Lysine	++
Arginine	++
Histidine	+++
Tyrosine	+
Tryptophan	+
3-Monochlorotyrosine	–
Monobromohistidine	++
Monobromotyrosine	+
Dichlorotyrosine	–
3-Monoiodotyrosine	–
Monobromo-Monochlorotyrosine	–
3,5-Dibromotyrosine	–
Monochloro-Monoiodotyrosine	–
Monobromo-Monoiodotyrosine	–
3,5-Diiodotyrosine	–

^1^ ++++ very strong decrease (residual content of 0%–25%); +++ strong decrease (residual content of 26%–50%); ++ medium decrease (residual content of 51%–75%); + weak decrease (residual content of more than 75%); − no decrease (residual 100% ± 15%).

In particular, the amount of threonine (peaks 10a, 10b) decreases very strongly. The amounts of AABA (peak 3), valine (peak 4), leucine (peak 5), phenylalanine (peak 11), aspartic acid (peak 12), and histidine (peak 17) also reduced considerably. For serine (peaks 6a, 6b), the decrease is different for the two TBDMS derivatives. The derivative with two TBDMS groups (incomplete derivatization) shows almost no decrease whereas the derivative with three TBDMS groups exhibits a medium decrease. This means, the total serine amount is slightly reduced. The same phenomenon is observed for hydroxyproline (peaks 9a, 9b). The amount of the derivative with three TBDMS groups strongly decreases whereas the amount of the derivative with two TBDMS groups (incomplete derivatization) only shows a medium decrease.

In contrast, the halogenated amino acids do not show a pronounced reduction, except for monobromohistidine (peak 21*). This observation leads to the conclusion that the halogenated tyrosines are strongly bound to the skeletons and confirms our hypothesis that halogenated molecules are tightly attached/covalently bound to the sponge skeleton.

Remarkably, traces of aerothionin or its derivatives can also be found in the skeletons after MeOH treatment. The identification was carried out as for the skeletons before MeOH treatment. This means, traces of such compounds are also incorporated into the skeletons of *A. cavernicola*. Also in this case, the retention time and the fragmentation pattern of peak B are consistent with those of the aerothionin standard.

#### 2.3.3. Analysis of the MeOH Extract

In the MeOH extracts, different non-halogenated amino acids could be detected by GC-MS. The detected amino acids were: alanine, glycine, valine, leucine, proline, serine, tyrosine, and 3,5-diiodotyrosine. Interestingly, threonine which was effectively removed by MeOH extraction is not detected in the MeOH extract. That means, a significant amount of the extracted molecules must be present in the form of peptides or smaller proteins—which will not be detected by the applied analytical method. Furthermore, aerothionin or its derivatives can be found in the MeOH extract, which confirms our previous results [28]. The identification was carried out as in the skeletons before MeOH treatment. The retention time and the fragmentation pattern of the found aerothionin fragment are consistent with those of the aerothionin standard. The mass spectrum of the found Aerothionin fragment is shown in the Supplementary Information (Figure S4).

### 2.4. LC-MS Analysis of the Skeletal Amino Acid Composition

#### 2.4.1. Skeletal Amino Acid Composition before MeOH Extraction

**Figure 7:**
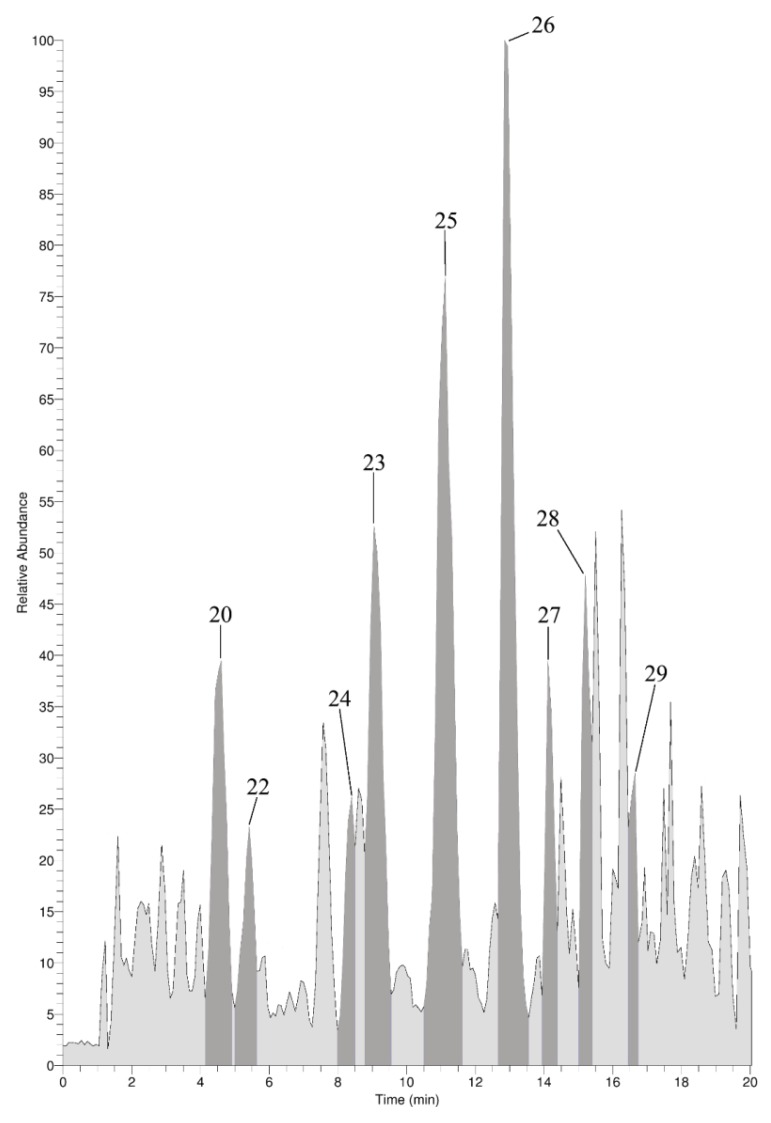
(+)-Ion trace of LC-MS analysis of the Ba(OH)_2_-extract prior MeOH extraction. Dark grey peaks correspond to halogenated tyrosines. Numbering refers to Table 1.

**Figure 8:**
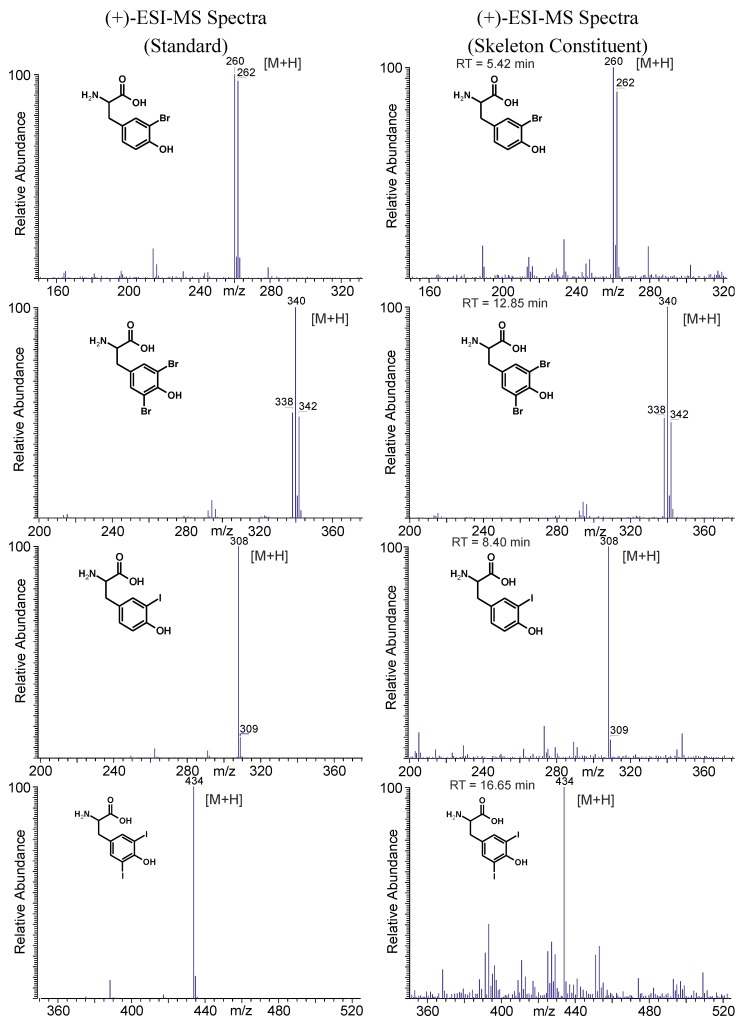
Structures and corresponding LC-ESI-MS spectra of brominated and iodinated tyrosine derivatives obtained from native standards and skeleton constituents of *A. cavernicola.*

In addition to the GC-MS measurements, an on-line LC-ESI-MS analysis was performed with the skeleton extract resulting from the Ba(OH)_2_ treatment. The focus of these measurements was placed on the verification of the presence of halogenated tyrosines. Since most of the other amino acids elute within the injection peak, they could not be determined via LC-MS. Apart from aliphatic hydroxyproline, ornithine, and arginine, the pseudo-molecular ions ([M + H]^+^) of aromatic tryptophan, phenylalanine, and tyrosine were detected (data not shown). For tyrosine, different halogenated derivatives were identified within the (+)-ion trace as shown in Figure 7. For monobromo-, 3,5-dibromo-, 3-monoiodo-, and 3,5-diiodotyrosine the identity of the skeleton constituents was established via retention time and mass spectra comparison with native standards as demonstrated in Figure 8. In addition, the corresponding [M + H]^+^-ions of mono- and dichloro-, as well as the mixed halogenated monobromo-monochloro-, monobromo-monoiodo- and monochloro-monoiodotyrosine were detected (spectra see Supplementary Information Figure S5).

#### 2.4.2. Skeletal Amino Acid Composition after MeOH Extraction

In analogy to 2.4.1, the Ba(OH)_2_ extracts of the skeletons after MeOH treatment were also analyzed by LC-MS. The halogenated tyrosines found before MeOH treatment remained present after extraction with the organic solvent (spectra see Supplementary Information Figure S6). Moreover, the MeOH extract of the *A. cavernicola* skeleton was also analyzed via LC-MS. A specific search for the pseudo-molecular ions of all the halogenated amino acids found in the Ba(OH)_2_ extracts was performed. None of the halogenated tyrosines identified in the Ba(OH)_2_ extracts were detectable in the MeOH extract. These observations confirm the aforementioned results of GC-MS (Subsections 2.3.1 and 2.3.2) which demonstrated that the halogenated tyrosines are strongly attached or incorporated into the skeletons.

## 3. Experimental Sections

### 3.1. Sponge Samples

The *A. cavernicola* samples were collected in the Mediterranean Sea by the Hydra-Institute at Elba, Italy [37] and purchased from the Hydra Institut für Meereswissenschaften AG (Munich, Germany). After underwater collection the sponge samples were shock frozen. The samples were transported and stored on dry ice.

### 3.2. Extraction of the Skeletons

#### 3.2.1. Isolation of the Skeleton

*A. cavernicola* skeletons were isolated from the sponge samples following the protocol described previously [28]. Approximately 10 g of the wet sponge were soaked in 40 mL of TE100 (10 mM Tris-HCl, 100 mM EDTA, pH 8). After two weeks, the remaining skeletons were washed in distilled water for 24 h under continuous shaking. The washing procedure was repeated six times.

#### 3.2.2. MeOH Extraction of the Skeletons

The MeOH extraction of the purified skeletons was performed with methanol and acetonitrile as described previously [28]. The isolated skeletons were transferred into 200 mL methanol and sonicated for 1 min. After 24 h, the solvent was renewed and the samples were stirred for a further 24 h. Afterwards one extraction cycle was performed in 200 mL acetonitrile. The extracted skeletons were freeze-dried. The extracts were combined and dried under a gentle stream of nitrogen.

#### 3.2.3. Ba(OH)_2_ Extraction

Thirty mg of each dried isolated skeleton before and after MeOH extraction was treated with 15 mL of saturated Ba(OH)_2_ solution containing 0.5 mg 5-bromotryptophan as internal standard at 37 °C for 6 days. Subsequently, the insoluble residue (chitin-fibers) was removed and the Ba(OH)_2_ solution was neutralized with H_2_SO_4_ and centrifuged. The supernatant was removed and freeze dried.

### 3.3. Derivatization

#### 3.3.1. Preparation of Standard Solutions

For the standard solutions, 12 mg of each amino acid was dissolved in a mixture of 5 mL H_2_O and 1 mL 2.5 M HCl. The solutions were stored in a refrigerator until analysis. As an internal standard, a solution containing 5-bromotryptophan was prepared following the same procedure.

#### 3.3.2. TBDMS-Derivatization of the Standard Solutions

Ten μL of the standard solution of the halogenated amino acids (alternatively 5 μL of the solution of the other amino acids) and 10 μL of the internal standard were mixed and dried under a gentle stream of nitrogen. The residue was soaked in 20 μL 2.5 M HCl and dried again. Subsequently, the residue was soaked twice in 40 μL EtOH and dried under nitrogen. 50 μL acetonitrile and 50 μL *N*-*tert*-butyldimethylsilyl-*N*-methylfluoroacetamide (MTBSTFA) with 1% *tert*-butyldimethylchlorosilane (TBDMSCl) were added to the dry residue. The mixture was sonicated for 30 s and then heated at 70 °C for 30 min. One μL of the resulting solution was injected into the GC-MS.

#### 3.3.3. TBDMS-Derivatization of the Sponge Samples

One mg of the dried Ba(OH)_2_ extract containing the internal standard was soaked in 20 μL 2.5 M HCl and dried under a gentle stream of nitrogen. Subsequently, the residue was soaked twice in 40 μL EtOH and dried under nitrogen. Fifty μL acetonitrile and 50 μL MTBSTFA were added to the dry residue. The mixture was sonicated for 30 s and then heated to 70 °C for 30 min. One μL of the resulting solution was injected into the GC-MS.

### 3.4. GC-MS Measurements

Analyses were carried out on a Varian 3400 gas chromatograph (Varian, Palo Alto, CA, USA) directly coupled to a Finnigan MAT 95 mass spectrometer (Finnigan/Thermo Fisher Scientific Inc., Waltham, MA, USA). The GC separations were performed on a SPB^®^-5 capillary GC column (Sigma-Aldrich, St. Louis, MO, USA). The flow of helium as carrier gas was 1 mL/min. The injector temperature was 300 °C. Split/Splitless injection was used (splitless time 1 min). The column temperature was programmed as followed: isothermal 115 °C for 3 min, then heating up to 300 °C at rate of 4K/min, then isothermal 300 °C for 30 min.

The ion source temperature was 250 °C and the transfer line temperature was 300 °C. The mass spectra were recorded with a scan cycle time of 1.284 s in the electron impact (EI) ionization mode at 70 eV, *m/z* range 70–850. The chromatograms were recorded after 3 min solvent delay. The retention time *t*_R_ was normalized to the second peak of the internal standard 5-bromotryptophan. The intensity was normalized to the weighted sample and the total intensity of both peaks of the internal standard.

### 3.5. Liquid Chromatography-Mass Spectrometry

#### 3.5.1. Preparation of Standard Solutions

Amino acids were dissolved in MeOH/H_2_O (10/1) to yield a sample concentration of 1 mg/mL.

#### 3.5.2. Preparation of Sponge Extract Samples

Dried extracts resulting either from the Ba(OH)_2_ treatment or the MeOH extraction of the *A. cavernicola* skeleton were dissolved in MeOH/H_2_O (10/1) to yield a sample concentration of 1 mg/mL.

#### 3.5.3. Measurement Conditions

All LC-MS analyses were performed on a Thermoquest Finnigan LCQDeca (Thermo Fisher Scientific Inc., Waltham, MA, USA) connected to an Agilent 1100 Series LC (Agilent, Santa Clara, CA, USA) with a Knauer Eurospher 100-3 C18, 100 × 2 mm column (Knauer, Berlin, Germany). As eluents 0.1% formic acid (Eluent A) and MeOH (Eluent B) were utilized and a gradient as shown in Table 5 was applied for all measurements. Electrospray ionization (ESI) mode was chosen for all analyzed samples.

**Table 5:** Eluent system utilized for LC-MS analyses.

*t* [min]	Eluent A [%]	Eluent B [%]
0	90	10
2	90	10
35	0	100
50	0	100
51	90	10
60	90	10

### 3.6. FTIR Spectroscopy

IR spectra were recorded using a Bruker FTIR spectrometer IFS 88 (Bruker, Karlsruhe, Germany). The samples were deposited on SPECAC Golden-Gate-ATR equipment (LOT-Oriel, Darmstadt, Germany). The spectra were measured in the range from 4000 to 650 cm^−1^ with a spectral resolution of 0.5 cm^−1^. Each spectrum was recorded by the accumulation of 1000 scans. Subsequently, an ATR intensity correction was carried out. The spectra were baseline corrected and normalized to the most intensive band at about 1625 cm^−1^.

### 3.7. Scanning Electron Microscopy (SEM) and Energy Dispersive X-ray Spectroscopy (EDX)

Some dried pieces of the skeletons were applied on a sample holder and coated with carbon. The SEM images were then recorded on a ZEISS DSM 982 GEMINI field emission scanning electron microscope (ZEISS, Oberkochen, Germany). Furthermore, EDX spectra were recorded. The spectra were measured with an acceleration voltage of 15 keV. The live time was 60 s.

### 3.8. Light Microscopy

Small pieces of the purified chitin-scaffolds after Ba(OH)_2_ or NaOH were put on a sample holder. Microscopic studies were carried out on a Keyence BZ-8000K microscope (Keyence, Osaka, Japan). The images were acquired at 10-fold magnification.

## 4. Conclusions

The isolated skeletons of the marine demosponge *A. cavernicola* before and after the conventional MeOH extraction were analyzed with respect to the presence of halogenated amino acids. For this purpose, a Ba(OH)_2_-based extraction method was applied which effectively removes all organic material from the remaining pure chitin. The Ba(OH)_2_ extracts of the sponge skeletons of *A. cavernicola* were studied by GC-MS and LC-MS. These investigations revealed that the skeletons contain 10 halogenated amino acids. Nine of these 10 amino acids are halogenated tyrosines. These halogenated tyrosines are strongly attached to the skeletons since the conventional MeOH extraction method does not significantly reduce the amount of halogenated tyrosines in the skeletons. Furthermore, 19 different non-halogenated amino acids were identified. Among them are 15 proteinogenic amino acids. Glycine and serine exhibit the largest concentrations, while leucine, phenylalanine, arginine, and tryptophan are less abundant. In addition, trace amounts of aerothionin and/or its derivatives are present.

To summarize, it should be emphasized that the identified halogenated tyrosines are integral parts of the chitin-based skeletons of *A. cavernicola*. It is tempting to speculate that these compounds are covalently bound to the skeletons. In contrast, the amount of non-halogenated organic material is reduced during MeOH treatment. Investigations of the MeOH extracts suggest that these removed molecules are not only free amino acids but also peptides or small proteins.

## Supplementary Files

Supplementary File 1
