# Supplementary material for: The Skeletal Amino Acid Composition of the Marine Demosponge Aplysina cavernicola
Source: Mar Drugs. 2014 Aug 8;12(8):4417–38. doi: 10.3390/md12084417 (PMC4145324; doi:10.3390/md12084417)
Supplement: Supplementary File 1 [file marinedrugs-12-04417-s001.pdf]

Supplementary Information

Figure S1. EDX measurement of the isolated skeletons of *Aplysina cavernicola* before MeOH extraction.

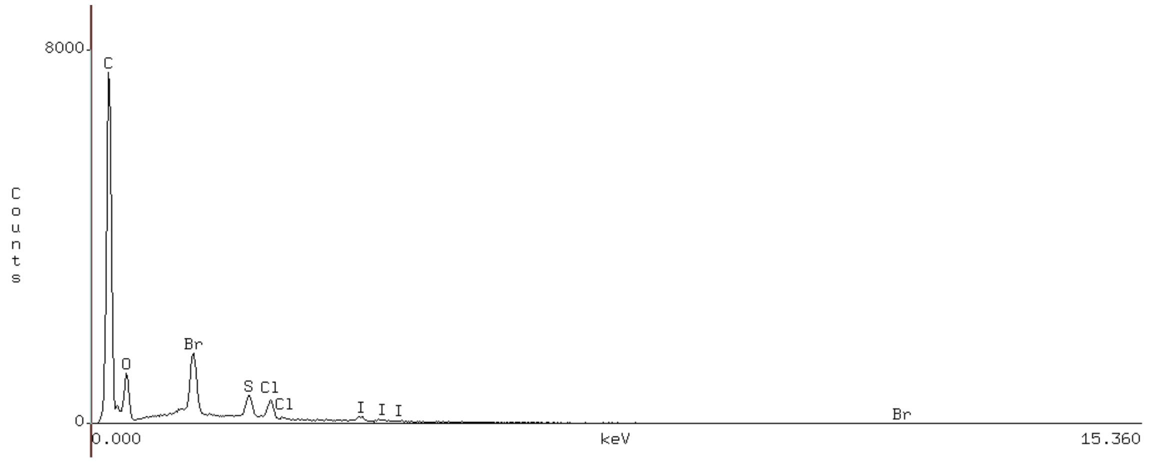

Figure S2. EDX measurement of the isolated skeletons of *A. cavernicola* after MeOH extraction.

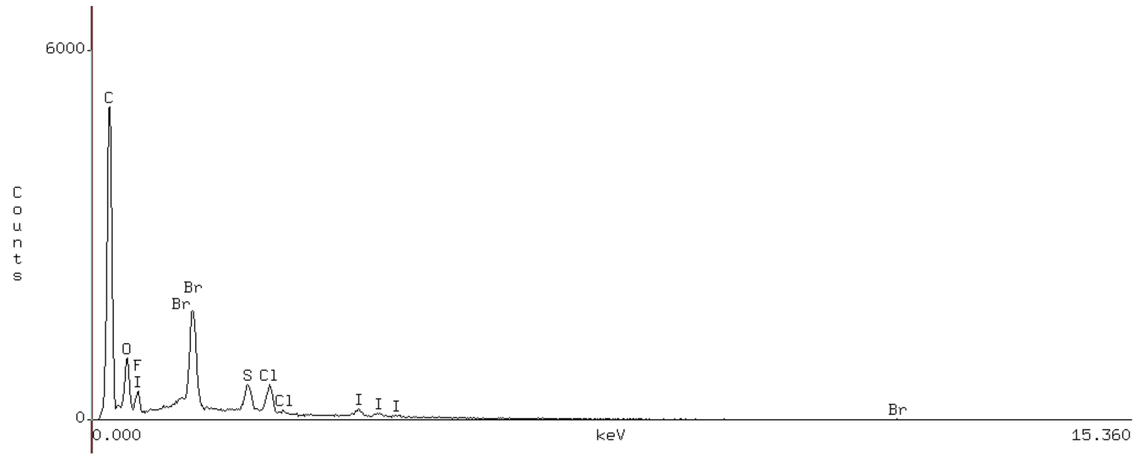

Figure S3. EI-MS data of TBDMS-derivative of the aerothionin standard.

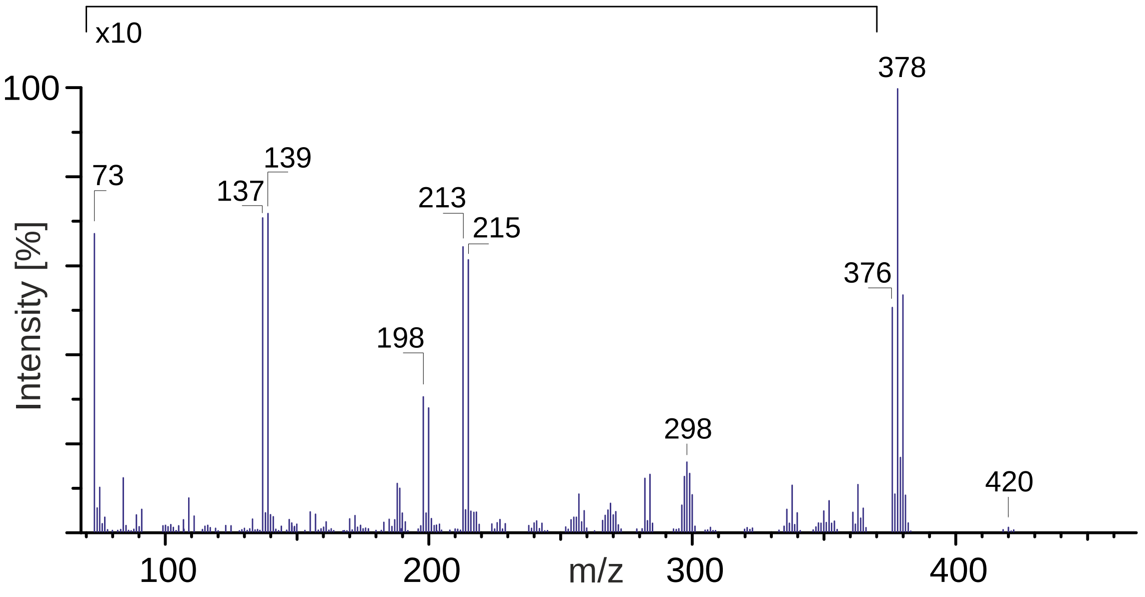

Figure S4. EI-MS data of TBDMS-derivative of the aerothionin fragment found in the MeOH extract.

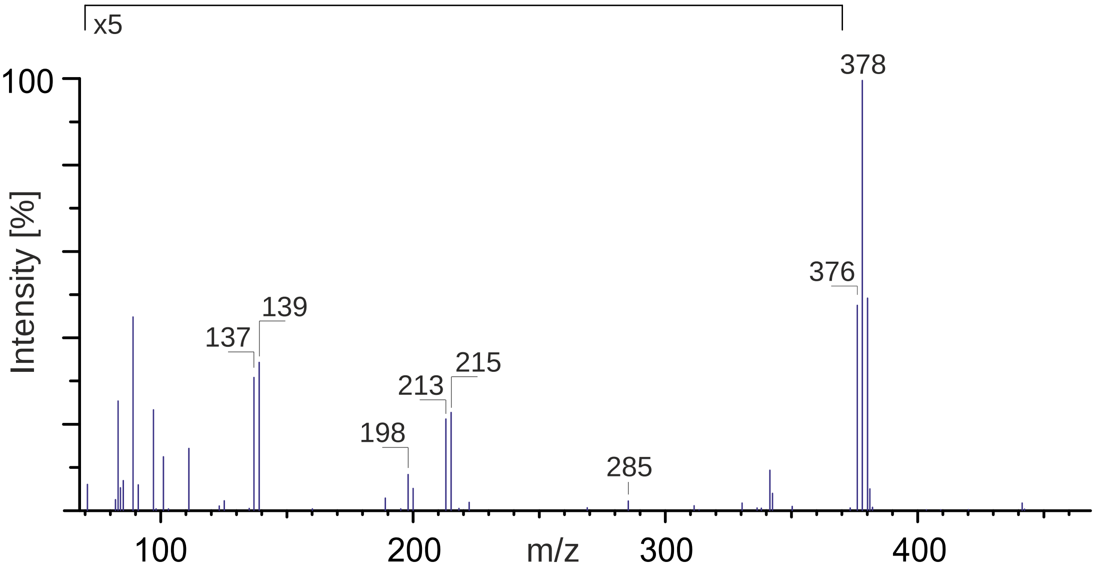

Table S1. Experimental Errors of six amino acids. * The experimental error is determined for these amino acids. For each amino acid, the experimental error is calculated from three measurements of the same sample. The average value is about 15%.

**Amino Acid**	**Experimental Error ***
Alanine (Peak 1)	4.8%
Phenylalanine (Peak 11)	23.8%
Aspartic Acid (Peak 12)	12.2%
Ornithine (Peak 14)	18.4%
Dichlorotyrosine (Peak 23*)	5.5%
Monobromo-Monochlorotyrosine (Peak 25*)	22.9%

Figure S5. LC-ESI-MS spectra of mono- and dichloro-, monobromo-monochloro-, monochloro-monoiodo- and monobromo-monoiodotyrosine detected in the $Ba(OH)_2$ skeleton extracts of *A. cavernicola*.

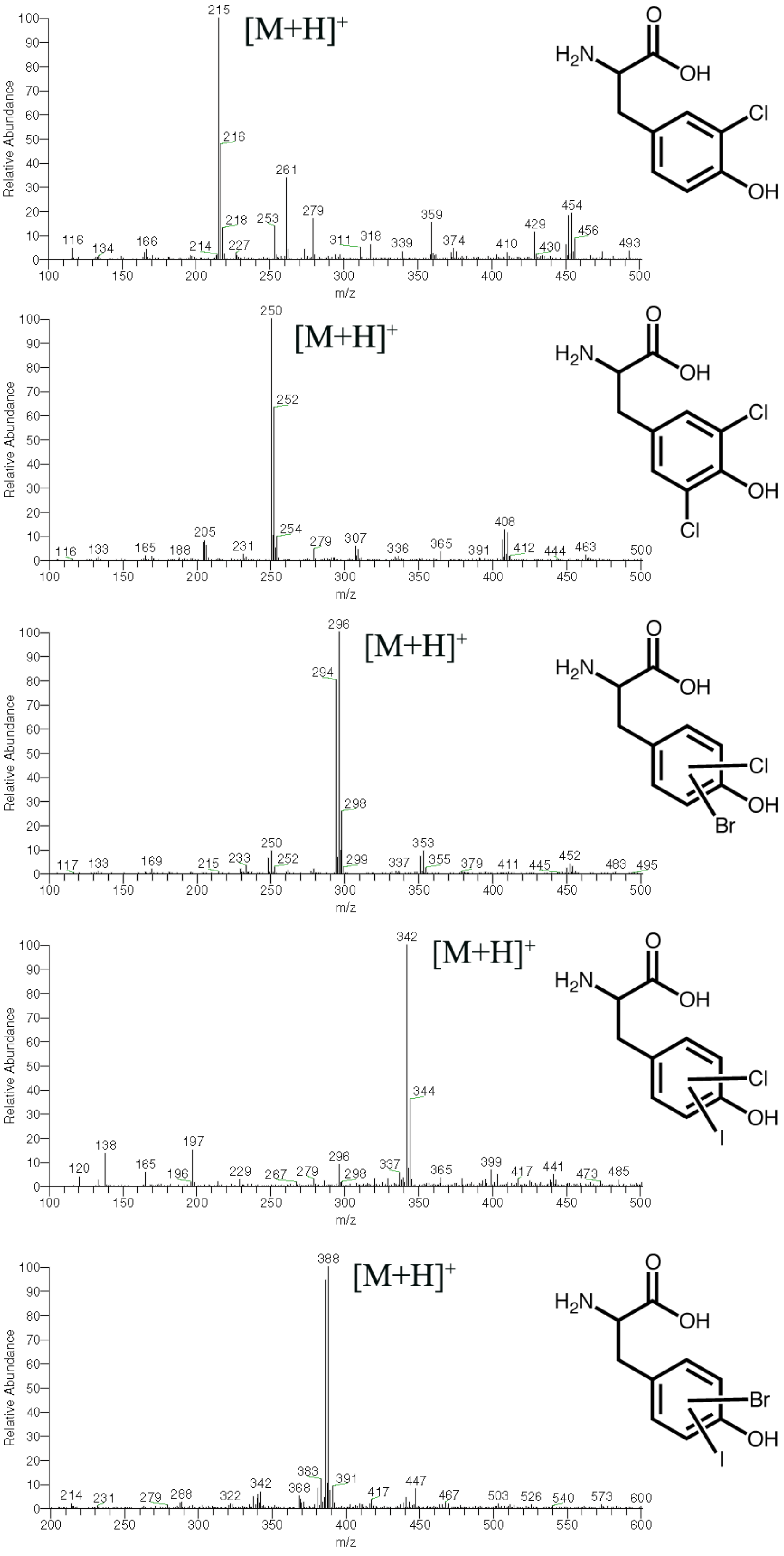

Figure S6. LC-ESI-MS spectra of halogenated tyrosines detected in the $Ba(OH)_2$ skeleton extract of *A. cavernicola* after MeOH extraction.

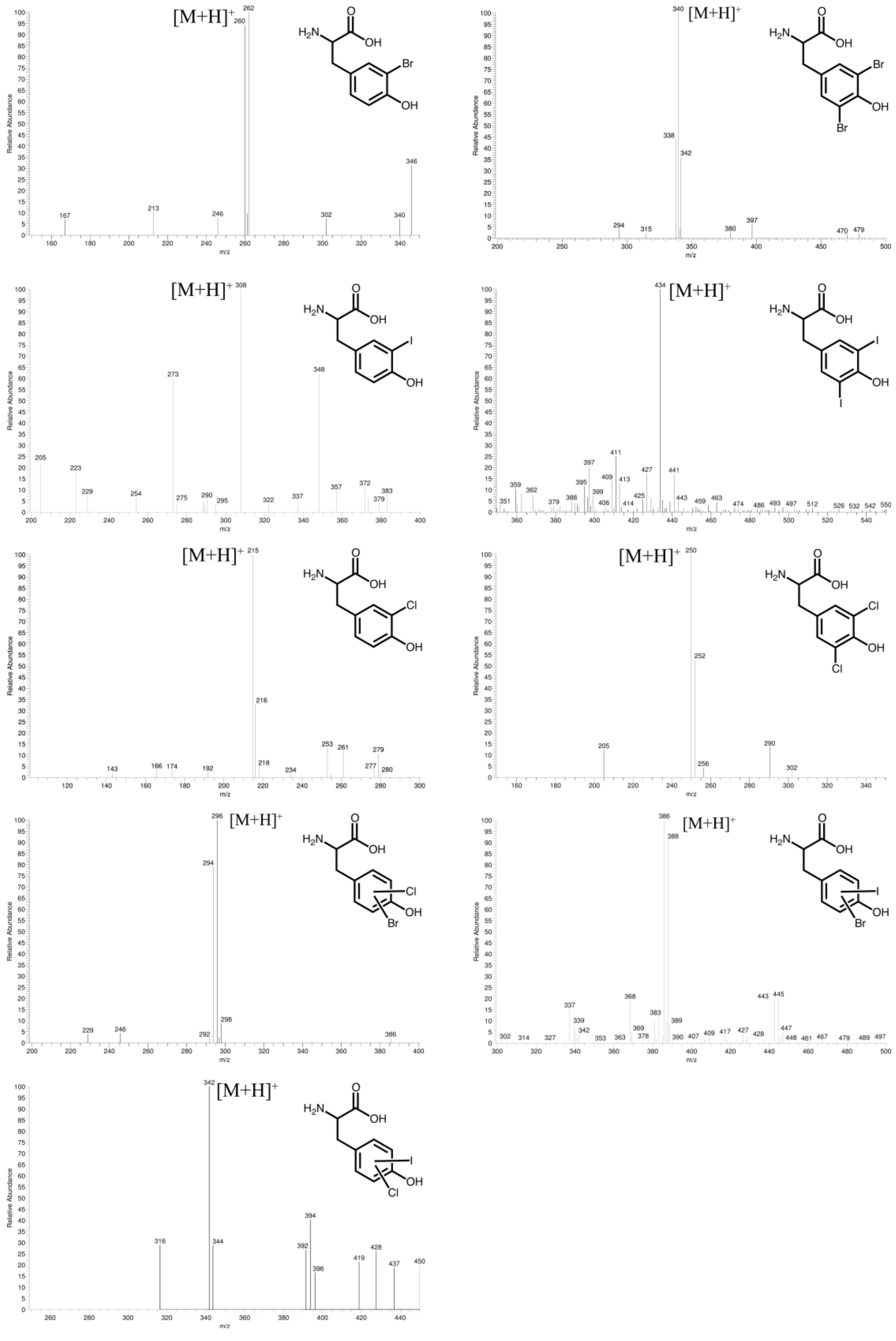

© 2014 by the authors; licensee MDPI, Basel, Switzerland. This article is an open access article distributed under the terms and conditions of the Creative Commons Attribution license (http://creativecommons.org/licenses/by/3.0/).
